# Hypocalcemia After Cervical Procedures in Patients with a History of Nonbariatric Gastrojejunostomy

**DOI:** 10.1245/s10434-025-18197-6

**Published:** 2025-09-05

**Authors:** Aviva S. Mattingly, Timothy Kravchenko, Sanjna Chokshi, Cindy Hakim, Jesse E. Passman, Sara Ginzberg, Solomiya Syvyk, Carrie E. Cunningham, Heather Wachtel, Douglas Fraker, Rachel Kelz, Lauren N. Krumeich

**Affiliations:** 1 Vanderbilt University, Nashville, TN USA; 2 University of Michigan, Ann Arbor, MI USA; 3 University of Pennsylvania, Philadelphia, PA USA; 4 Massachusetts General Hospital, Boston, MA USA

## Abstract

**Background:**

Hypocalcemia is common after cervical procedures. Patients who have undergone Roux-en-Y gastric bypass (RYGB) experience increased risk for post-thyroidectomy hypocalcemia. This association has not been elucidated for nonbariatric operations that bypass the duodenum.

**Methods:**

A multi-institutional retrospective cohort study included patients who underwent parathyroidectomy and/or thyroidectomy with prior sleeve gastrectomy (SG), bariatric RYGB, or nonbariatric gastrojejunostomy (GJ). The primary outcomes were early (≤6 months) and late (>6 months) postoperative hypocalcemia. The secondary outcomes were prolonged length of stay (>24 hours) and 30-day readmission.

**Results:**

A total of 241 patients had prior SG (39%), RYGB (44%), or GJ (17%). Early (54%) and late (41%) hypocalcemia were common. Patients with prior GJ compared with SG had significantly higher rates of early hypocalcemia (64% vs. 44% *p* = 0.04). The rate of late hypocalcemia was higher in those with prior GJ (53%, *p* = 0.007) or RYGB (49%, *p* = 0.003) compared with SG (28%). By multivariable regression, early hypocalcemia was positively associated with parathyroid autotransplantation (odds ratio [OR] 6.36, *p* = 0.005), and more parathyroid glands removed (OR 1.45, *p* = 0.03), while higher preoperative calcium was associated with lower odds of hypocalcemia (OR 0.51, *p* = 0.02). Late hypocalcemia was independently associated with RYGB (OR 2.38, *p* = 0.01) and GJ (OR 3.1, *p* = 0.01). The highest rates of early (81%) and late (71%) hypocalcemia were among those with prior nonbariatric GJ who underwent total thyroidectomy. Early hypocalcemia was associated with prolonged length of stay and 30-day readmission.

**Conclusions:**

Patients with prior GJ or RYGB frequently experience hypocalcemia following cervical procedures, informing preoperative counseling and perioperative management. Preoperative calcium optimization is a potential mitigative strategy warranting further study.

Hypocalcemia is a common postoperative occurrence after total thyroidectomy or parathyroidectomy. The reported incidence varies in the literature based on institutional parameters and the criteria used for the definition, with overall hypocalcemia rates ranging from 17% to 44%.^1–7^ Hypocalcemia can be related to anatomic factors, such as parathyroid removal or vascular disruption, as well as patient factors, such as rapid resorption of calcium into bone or deficits in calcium absorption. Common symptoms of hypocalcemia include numbness and tingling around the mouth and in the digits. Although rare, profound hypocalcemia can potentiate tetany, seizures, and cardiac arrythmias, so patients with symptomatic hypocalcemia warrant hospitalization for monitoring and calcium administration.^8–10^ With awareness of the condition and appropriate surveillance and supplementation, clinicians can manage most instances of postoperative hypocalcemia outpatient with short-term calcium supplementation and calcitriol.^3,11,12^ Understanding risk factors of postoperative hypocalcemia can facilitate appropriate counseling and management of this common and potentially dangerous condition.

Intestinal calcium absorption takes place through two mechanisms that are determined by anatomy. The more effective mechanism is a vitamin-D–dependent transcellular process that predominantly occurs in the duodenum and upper jejunum. In contrast, the paracellular mechanism is concentration-dependent and occurs throughout the length of the intestine.^13^ Patients who have undergone bariatric surgery involving duodenal bypass, such as Roux-en-Y gastric bypass (RYGB) or biliopancreatic diversion, experience increased risk for recalcitrant post-thyroidectomy hypocalcemia and symptomatic hypocalcemia requiring intravenous supplementation.^14–19^ Hypocalcemia after RYGB is suspected to be predominantly secondary to the loss of the vitamin-D–dependent transcellular process. Calcium citrate is preferred in this setting owing to its superior paracellular absorption and less dependency on pH for absorption.^20^

Patients undergoing cervical procedures may have a history of gastrojejunostomy (GJ) in the setting of other procedures, such as pancreaticoduodenectomy (Whipple procedure), total pancreatectomy, or distal gastrectomy. It has not yet been thoroughly studied if there is an increased risk of hypocalcemia following total thyroidectomy and/or parathyroidectomy in patients with a history of nonbariatric GJ. Whereas the average roux limb in a RYGB is greater than 100 cm,^21^ a GJ performed for nonbariatric purposes is as short as anatomically feasible. However, because the duodenum is the predominant location of calcium absorption and remains anatomically excluded in patients who have undergone short segment nonbariatric GJ, we hypothesized that they would also demonstrate an increased risk for hypocalcemia after total thyroidectomy and/or parathyroidectomy. We hypothesized that these patients would also experience increased length of stay (LOS) and 30-day readmission.

## Methods

### Study Population and Variables

We performed a multi-institutional retrospective cohort study of patients at four tertiary medical centers (University of Pennsylvania, Massachusetts General Hospital, Brigham and Women’s Hospital, University of Michigan) who presented for parathyroidectomy with or without concurrent thyroid surgery or total/completion thyroidectomy in isolation who had undergone either: 1) sleeve gastrectomy (SG), 2) bariatric RYGB, or 3) short segment nonbariatric GJ reconstruction following a Whipple procedure, total pancreatectomy, or distal gastrectomy prior to the cervical operation. Patients with a prior SG were used as a comparative group given that there is no intestinal (duodenal) bypass involved. Patients who underwent the cervical procedures between 2003 and 2024 were included. Manual chart review was performed to obtain clinical variables potentially associated with hypocalcemia, including demographics, medication use, laboratory values, operative details, comorbidities, and surgical history. Each institution obtained institutional review board approval with a waiver of consent.

Patients were excluded if they had no postoperative labs within 1 year of the cervical operation (N = 14) or if the dates of the operations could not be confirmed (N = 5) to ensure that the abdominal procedure preceded the cervical procedure. Patients were not excluded if they had a history of colectomy based on the pathophysiology of calcium absorption in the small intestine. No patients had undergone additional enterectomy.

### Definitions and Outcomes

Preoperative labs within 1 year of the cervical operation were evaluated. The primary outcomes were early (≤6 months) and late (>6 months) postoperative hypocalcemia, defined by institutional laboratory parameters that ranged from 8.4 to 8.8 mg/dL. Hypocalcemia was corrected for hypoalbuminemia when albumin was recorded within 30 days of the calcium measurement. The secondary outcomes were prolonged LOS (>24 hours) and 30-day readmission. Whether a parathyroid autotransplant was performed was determined by the operative reports. The number of parathyroid glands removed was confirmed by surgical pathology reports. Autotransplanted gland(s) and intraoperative parathyroid biopsies were not included in this figure. Calcium supplementation was calculated based on the amount of elemental calcium and the type of calcium administered, using 40% of calcium carbonate dosing and 21% of calcium citrate to reflect the elemental portion.

### Statistics

The Skewness and Kurtosis test was used to define normality. To evaluate differences between groups, we utilized Kruskal-Wallis tests for continuous nonparametric data and chi-square tests to compare independent proportions. The Fisher’s exact test was utilized when cell counts were less than five. We prospectively intended to perform subgroup analysis by the type of cervical procedure. Backward stepwise logistic regression was used to identify variables independently correlated with a risk of the primary outcomes (early and late hypocalcemia). Based on the logistic regression results, a receiver operating characteristic curve was performed for continuous variables of interest to assess for their predictive value in discriminating postoperative hypocalcemia. Percentages in results tables are reported based on the number of patients with available data.

## Results

### Patient Characteristics

This study included 241 patients with a median age of 57 (interquartile range [IQR] 49-65). Most of the cohort was female (85%) and Caucasian (74%). Regarding history of gastrointestinal surgery, 95 (39%) had a history of SG, 106 (44%) RYGB, and 40 (17%) nonbariatric GJ (28 following a Whipple procedure or total pancreatectomy and 12 following a distal gastrectomy) (Table 1). The median interval between the abdominal and cervical operations was about 48 months (IQR 17–86).

**Table 1:** Demographic characteristics of study population (N = 241)

Characteristics	N (%)^a^ or median (IQR)
Median age, years	57 (49–65)
Female	204 (85)
BMI, kg/m^2^	33 (29–38)
*Race*	
Caucasian	178 (74)
African American	50 (21)
Hispanic	2 (0.8)
Mixed race/other	7 (3)
Unknown	4 (2)
*Preoperative labs (units)*	
Albumin (g/dL)	4.2 (4.0–4.4)
Calcium (mg/dL)	9.7 (9.3–10.3)
Creatinine (mg/dL)	0.77 (0.65–0.90)
OH-vitamin D (ng/mL)	34 (27–45)
Parathyroid hormone (pg/mL)	89 (59–133)
Magnesium (mg/dL)	2.0 (1.8–2.1)
*Abdominal procedure*	
Sleeve gastrectomy	95 (39)
Roux-en-Y gastric bypass	106 (44)
Distal gastrectomy with gastrojejunostomy	12 (5)
Whipple or total pancreatectomy	28 (12)
Months between abdominal and cervical procedures	48 (17–86)
*Cervical procedure*	
Parathyroidectomy	102 (42)
Total or completion thyroidectomy	127 (53)
Parathyroidectomy with partial or total thyroidectomy	12 (5)
*Postoperative medications*	
Proton pump inhibitor	90 (39)
H2 blocker	23 (10)
Calcium carbonate	144 (61)
Calcium citrate	77 (33)
No calcium supplementation	13 (6)
*Postoperative labs (within 7 days)*	
Parathyroid hormone (pg/mL)	28 (13–45)
Calcium (mg/dL)	9 (8.1–9.4)
*Outcomes*	
Early hypocalcemia	120 (54)
Late hypocalcemia	93 (41)
Length of stay (hours)	23 (8–28)
Prolonged length of stay >24 hours	94 (42)
30-day readmission	24 (11)

^a^Percentages are reported among patients with available data

During the observed encounter, most patients underwent total or completion thyroidectomy (53%) or parathyroidectomy (42%); a small portion underwent a combination of parathyroidectomy with partial or total thyroidectomy (5%). For those who underwent parathyroidectomy, 51% underwent a single gland removal, and the rest had more than one gland removed (median 1; IQR 1–3). Sixteen percent of patients who underwent thyroidectomy received a parathyroid autotransplantation, and 11% incidentally had a parathyroid removed with the thyroid specimen. Among the thyroidectomy patients who required parathyroid autotransplantation, 32% had undergone a concurrent central neck dissection. The median parathyroid hormone (PTH) level after parathyroidectomy was 26 pg/mL (IQR 15–49) compared with 28 pg/mL (IQR 12–46) after exclusive thyroidectomy (*p* = 0.2). The median preoperative calcium for patients undergoing thyroidectomy was 9.5 mg/dL (IQR 9.2–9.7); fewer than 10% of patients undergoing thyroidectomy had preoperative hypocalcemia.

Approximately half of patients were documented as being prescribed either a proton pump inhibitor (N = 90, 39%) or H2 antagonist (N = 23, 10%) preoperatively. The documented rate of proton pump inhibitor use was 60% after prior nonbariatric GJ, 39% after prior RYGB, and 32% after prior SG (Table 2).

**Table 2:** Patient characteristics based on extent of intestinal resection/bypass

Characteristics	GJ (N = 40)	RYGB (N = 106)	SG (N = 95)	*p*
Median age, years, *median (IQR)*	65 (53–73)	56 (49–63)	55 (46–63)	**0.007**
Female, *N (%)*	33 (83)	94 (87)	77 (81)	0.3
BMI, kg/m^2^, *median, (IQR)*	26 (23–33)	32 (29–38)	35 (31–38)	**<0.001**
*Race, N (%)*				
Caucasian	31 (78)	79 (75)	68 (72)	0.28
African American	7 (18)	18 (17)	25 (26)	
Hispanic	1 (2.5)	1 (1)	0 (0)	
Mixed race/other	0 (0)	5 (5)	2 (2)	
Unknown	1 (2.5)	3 (3)	0 (0)	
*Preoperative labs, median (IQR)*				
Albumin	4.2 (3.8–4.5)	4.2 (4.0–4.4)	4.2 (4.0–4.4)	0.54
Calcium	9.8 (9.3–10.6)	9.6 (9.2–10.2)	9.7 (9.4–10.3)	0.25
Creatinine	0.80 (0.66–0.90)	0.73 (0.62–0.84)	0.80 (0.67–0.97)	**0.02**
OH-vitamin D	30 (22–47)	33 (22–43)	35 (30–45)	0.24
Parathyroid hormone	90 (75–165)	104 (60–142)	77 (54–100)	0.15
Magnesium	1.9 (1.9–2.0)	1.9 (1.8–2.0)	2.0 (1.8–2.1)	0.84
*Cervical procedure, N (%)*				
Parathyroidectomy	18 (45)	47 (44)	37 (39)	0.09
Total or completion thyroidectomy	17 (43)	54 (51)	56 (59)	
Parathyroidectomy + partial or total thyroidectomy	5 (13)	5 (5)	2 (2)	
Months between procedures, *median (IQR)*	31 (10–67)	59 (22–114)	30 (13–63)	**<0.001**
*Postoperative medications, N (%)*				
Proton pump inhibitor	21 (60)	39 (39)	30 (32)	**0.017**
H2 blocker	5 (15)	10 (10)	8 (9)	0.6
Calcium carbonate	20 (50)	72 (68)	52 (55)	0.06
Calcium citrate	15 (38)	26 (25)	36 (38)	0.09
*Postoperative labs (within 7 days, median (IQR))*				
Parathyroid hormone	32 (12–42)	26 (12–46)	28 (18–45)	0.8
Calcium	9.1 (8.6–9.4)	8.8 (8.0–9.2)	9 (8.1–9.5)	0.2
*Postoperative outcomes, N (%)*				
Early hypocalcemia	25 (64)	57 (58)	38 (44)	0.07
Late hypocalcemia	20 (53)	49 (49)	24 (28)	**0.004**
Length of Stay (hours), *median (IQR)*	28 (23–48)	23 (8-27)	22 (6–27)	**0.002**
Prolonged length of stay	23 (66)	35 (37)	36 (39)	**0.009**
30-day readmission	7 (26)	7 (7)	10 (12)	**0.02**
Readmission for hypocalcemia	2 (8)	2 (2)	7 (9)	0.11

Only 31% of patients had 25-OH vitamin D levels measured preoperatively, with a median level of 34 ng/mL (IQR 27–45). Preoperatively, 24% and 33% of patients had documented calcium and vitamin D supplements, respectively. With one exception, all patients on preoperative calcium supplementation lacked hypocalcemia prior to the cervical procedure. After surgery, all but 6% of patients were documented as being prescribed calcium supplementation. The majority received calcium carbonate (61%) or calcium citrate (33%). The median daily elemental calcium received was 600 mg (IQR 336–900), regardless of intestinal anatomy. Liquid calcium was rarely administered (N = 2). Vitamin D was often added to the postoperative supplementation (78%) either combined within the calcium supplement or in the form of calcitriol.

### Early and Late Hypocalcemia

**Fig. 1:**
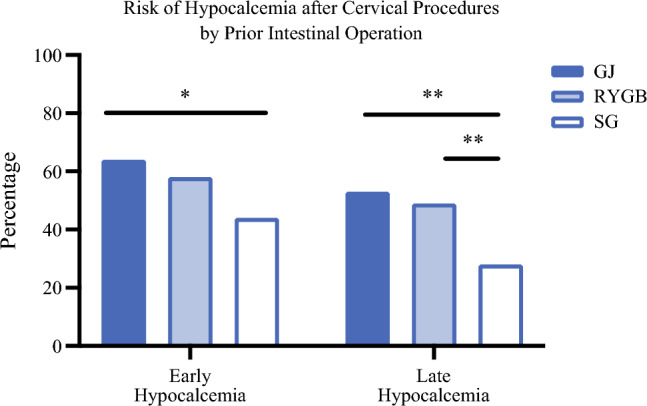
Incidence of early (≤6 months) or late (>6 months) postoperative hypocalcemia after total thyroidectomy and/or parathyroidectomy by prior intestinal operation (*p < 0.05; **p < 0.01). *GJ* short segment gastrojejunostomy; *RYGB* bariatric Roux-en-Y gastric bypass; *SG* bariatric sleeve gastrectomy

Early hypocalcemia was exceedingly common within this cohort, experienced by 120 patients (54%). The rates of early hypocalcemia were 44% after prior SG, 58% after prior RYGB, and 64% after prior nonbariatric GJ (Fig. 1). The difference was significantly higher after nonbariatric GJ (*p* = 0.04) but not RYGB (*p* = 0.07) compared with SG. By multivariable regression, factors positively associated with early hypocalcemia included parathyroid autotransplantation (OR 6.36, *p* = 0.005), more parathyroid glands removed (OR 1.45, *p* = 0.03), and higher postoperative elemental calcium supplementation (OR 1.56 for each 500 mg prescribed daily, *p* = 0.008), while higher preoperative calcium level was associated with lower odds of early hypocalcemia (OR 0.51, *p* = 0.02) (Table 3). While nonbariatric GJ anatomy was associated with early hypocalcemia on univariate analysis, it did not reach significance when adjusted for other factors in the multivariable model.

**Table 3:** Multivariable logistic regression for *early* postoperative hypocalcemia

Characteristic	Univariate				Multivariable			
	β	OR	95% CI	*p*	β	OR	95% CI	*p*
Age	−0.02	0.98	0.96–1.00	0.06				
Female gender	−0.03	0.97	0.47–0.99	0.93				
*Race*								
Caucasian	Ref	–	–	–				
African American	−0.37	0.69	0.36–1.34	0.3				
Hispanic	–	–	–	NS				
Other	–	–	–	NS				
Central node dissection	0.61	1.83	0.71–4.75	0.21				
Preoperative serum calcium	−0.56	0.57	0.39–0.82	**0.003**	−0.67	0.51	0.29–0.91	**0.02**
Preoperative vitamin D (total)	−0.03	0.97	0.93–1.00	0.07				
Final postoperative parathyroid hormone (≤7 days)	0.001	1.00	0.99–1.01	0.82				
Postoperative antacid	0.42	1.52	0.88–2.63	0.13				
Postoperative vitamin D supplementation	0.83	2.30	1.08–4.86	**0.03**				NS
*Postoperative calcium supplementation*^a^								
Calcium carbonate	1.76	5.82	1.21–27.97	**0.03**				NS
Calcium citrate	1.64	5.16	1.04–25.56	**0.04**				NS
Calcium gluconate	–	–	–	NS				
Postop elemental Ca^b^	<0.01	1.49	1.15–1.92	**0.002**	<0.01	1.56	1.15–2.21	**0.008**
*Prior intestinal operation*								
Sleeve gastrectomy	Ref	–	–	–	Ref	–	–	–
Gastrojejunostomy	0.81	2.26	1.03–4.92	**0.04**	0.80	2.23	0.83–5.94	0.11
Roux-en-Y gastric bypass	0.54	1.71	0.96–3.07	0.07	0.70	2.02	0.96–4.25	0.07
*Operative details*								
# Parathyroid glands autotransplanted	1.88	6.58	1.83–23.69	**0.004**	1.84	6.36	1.75–23.12	**0.005**
# Parathyroid glands removed	0.19	1.2	0.95–1.53	0.1	0.37	1.45	1.03–2.05	**0.03**

^a^Reference:no calcium supplementation

^b^Odds ratio for every 500 mg daily

Late hypocalcemia was also frequent in this cohort, experienced by 93 patients (41%). The rate of late hypocalcemia was higher in both those with prior RYGB (49%, *p* = 0.003) or nonbariatric GJ (53%, *p* = 0.007) compared with SG (28%). In the multivariable model, late hypocalcemia was associated RYGB anatomy (OR 2.38, *p* = 0.01) and nonbariatric GJ anatomy (OR 3.1, *p* = 0.01) and remained associated with parathyroid autotransplantation (OR 2.78, *p* = 0.03), number of parathyroid glands removed (OR 1.35, *p* = 0.04), and preoperative calcium level (OR 0.49, *p* = 0.002). Preoperative elemental calcium supplementation, vitamin D supplementation, formulation of postoperative calcium supplementation, use of vitamin D supplementation, and postoperative PTH were not significant in either multivariable model.

Receiver operating characteristic curves were performed to assess for the accuracy of preoperative calcium level in predicting early and late postoperative hypocalcemia. The area under the curve for preoperative calcium level was 64% and 63% for early and late hypocalcemia, respectively. A preoperative calcium level of 9.3 mg/dL maximized specificity (early hypocalcemia: 89%, late hypocalcemia: 87%) and sensitivity (both: 31%) for postoperative hypocalcemia. A threshold of <8.8 mg/dL was highly specific (>97%) for any postoperative hypocalcemia. These values were comparable when exclusively evaluating thyroidectomy patients.

### Subgroup Analysis of Hypocalcemia by Cervical Procedure Type

**Fig. 2:**
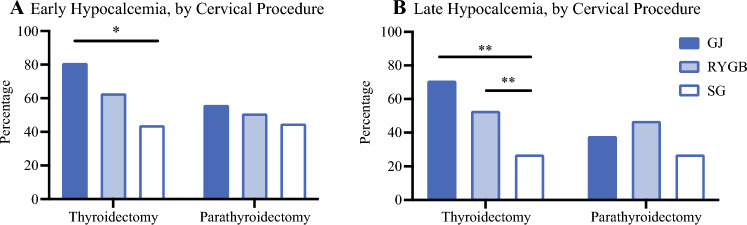
Incidence of early (≤6 months) or late (≥6 months) postoperative hypocalcemia after total thyroidectomy and/or parathyroidectomy by prior intestinal operation, divided by cervical operation. Patients who underwent both thyroidectomy and parathyroidectomy were excluded from this subanalysis (*p < 0.05, **p < 0.01). *GJ* short segment nonbariatric gastrojejunostomy; *RYGB* bariatric Roux-en-Y gastric bypass; *SG* bariatric sleeve gastrectomy

The highest rate of postoperative hypocalcemia was seen in the subgroup of patients undergoing thyroidectomy. In this group, the rate of early hypocalcemia reached 81% for those with prior nonbariatric GJ and 63% for those with RYBG, which were both higher than those with prior SG (44%, *p* = 0.01,* p* = 0.06 respectively). Similarly, the rates of late hypocalcemia were 71% after prior nonbariatric GJ (*p* = 0.001) and 53% after prior RYGB (*p* = 0.007) compared with a late hypocalcemia rate of 27% in those with prior SG (Fig. 2). As expected, those with preoperative hypocalcemia had high rates (80%) of postoperative hypocalcemia across the cohort. However, even when only analyzing those with normocalcemia preoperatively, the rates of hypocalcemia among those with prior GJ undergoing total thyroidectomy remained 77% (early) and 64% (late). In comparison, the rates of early and late hypocalcemia for those undergoing parathyroidectomy were not statistically different based on gastrointestinal procedure (Fig. 2).

By multivariable regression among the subgroup of thyroidectomy patients, GJ anatomy was associated with 5.5 times higher odds (95% CI 1.52–20.23, *p* = 0.01) and RYGB anatomy was associated with 3.2 times higher odds (95% CI 1.22–8.38, *p* = 0.02) of late hypocalcemia. Preoperative calcium, parathyroid autotransplantation, and number of parathyroid glands removed were not independent factors in this model.

### Length of Stay

The median LOS was 23 hours (IQR 8–28). Ninety-four patients (42%) had a prolonged LOS, more commonly following a total/completion thyroidectomy (61%) compared with parathyroidectomy (33%) or combined thyroidectomy and parathyroidectomy (6%). Prolonged LOS was more common in those with prior GJ (66%) compared with SG (39%, *p* = 0.006) or RYGB (37%, *p* = 0.003) (Table 2). Prolonged LOS was associated with early (*p* = 0.008) but not late (*p* = 0.26) hypocalcemia. The median LOS for those with early hypocalcemia was 25 hours (IQR 21–48).

### Readmission

The 30-day readmission rate was 11% (N = 24). Approximately half of these readmissions were due to hypocalcemia (N = 11). The rate of 30-day readmission was significantly different across gastrointestinal procedure groups (*p* = 0.02). The highest 30-day readmission was seen in patients with a history of GJ (26%) compared with SG (12%) and RYGB (7%) (Table 4). Although there was a wide variety of reasons for readmission, including gastrointestinal complaints, cardiac symptoms, and infection, when hypocalcemia was present, the rate of readmission was higher compared with when there was no early hypocalcemia (16% vs. 7%, *p* = 0.05).

**Table 4:** Multivariable logistic regression for *late* postoperative hypocalcemia

Characteristic	Univariate				Multivariable			
	β	OR	95% CI	*p*	β	OR	95% CI	*p*
Age	−0.01	1.00	0.97–1.01	0.25				
Female gender	−0.16	0.85	0.42–1.75	0.66				
*Race*								
Caucasian	Ref	–	–	–				
African American	−0.04	0.96	0.5–1.84	0.90				
Hispanic	–	–	–	–				
Other	–	–	–	–				
Central node dissection	0.21	1.24	0.51–3.02	0.64				
Preoperative serum calcium	−0.67	0.51	0.35–0.75	**<0.001**	− 0.71	0.49	0.31–0.77	**0.002**
Preoperative vitamin D (total)	−0.02	0.98	0.94–1.02	0.23				
Final postoperative parathyroid hormone (≤7 days)	0.01	1.01	0.99–1.02	0.20				
Postoperative antacid	0.26	1.30	0.75–2.25	0.35				
Postoperative vitamin D supplementation	0.98	2.66	1.19–5.94	**0.02**				NS
*Postoperative calcium supplementation*^a^								
Calcium carbonate	0.84	2.32	0.47–11.34	0.30				
Calcium citrate	1.53	4.61	0.91–23.23	0.06				
Calcium gluconate	–	–	–	NS				
Postop elemental Ca ^b^	<0.01	1.2	1.01–1.43	**0.04**				NS
*Prior intestinal operation*								
Sleeve gastrectomy	Ref	–	–	–	Ref	–	–	–
Gastrojejunostomy	1.07	2.92	1.32–6.44	**0.008**	1.13	3.10	1.27–7.56	**0.01**
Roux-en-Y gastric bypass	0.91	2.45	1.34–4.56	**0.004**	0.87	2.38	1.19–4.75	**0.01**
*Operative details*								
# Parathyroid glands autotransplanted	0.92	2.52	1.04–6.08	**0.04**	1.02	2.78	1.11–6.99	**0.03**
# Parathyroid glands removed	0.13	1.14	0.89–1.45	0.3	0.3	1.35	1.02–1.78	**0.04**

^a^Reference:  no calcium supplementation

^b^Odds ratio for every 500 mg daily

## Discussion

In this study, we found that early and late postoperative hypocalcemia are exceedingly common after cervical procedures in patients with histories of foregut bypass. Consistent with our hypothesis, a history of duodenal bypass, either through nonbariatric short segment GJ or bariatric RYGB, predicted late hypocalcemia more so than SG. SG was used as the comparator to control for a gastric procedure that did not include duodenal bypass to isolate the effect of the bypass. As expected, the rate of hypocalcemia in the SG group was higher than the 22% rate reported in patients without any medical or surgical malabsorption condition.^18^ While SG is a restrictive bariatric procedure, higher rates of early hypocalcemia in SG could be related to calcium malabsorption due to accelerated gastric emptying and intestinal motility or a reduction in stomach acid.^22^ The increased risk of hypocalcemia in the bypass groups suggests factors beyond those experienced after SG, which we hypothesize is the duodenal bypass itself. The group at highest risk for early and late hypocalcemia were those with a history of GJ undergoing total thyroidectomy. This subgroup analysis highlights the potential for focused intervention.

When considering other preoperative risk factors for hypocalcemia, calcium level among thyroidectomy patients was a significant predictor of both early and late hypocalcemia. Higher preoperative calcium levels were associated with lower rates of postoperative hypocalcemia. Even patients with low to normal calcium (<9.3 mg/dL) preoperatively were at risk for postoperative hypocalcemia. Preoperative hypocalcemia should cue providers to patients at higher risk for postoperative hypocalcemia, and preoperative normocalcemia should not necessarily be reassuring. Given the low rates and amount of preoperative supplementation identified in this multi-institutional evaluation, we highlight preoperative calcium supplementation as an area of possible intervention to investigate prospectively in this high risk population.

This study also explored intraoperative factors associated with hypocalcemia. Parathyroid autotransplantation was associated with early and late hypocalcemia, with a more pronounced impact on the odds of early hypocalcemia. This may suggest that reimplanted glands when not yet functional put patients at higher risk of hypocalcemia in the short term, but regain some function within 6 months postoperatively. This is consistent with prior findings of transient hypocalcemia following autotransplantation, despite the role for protection against permanent hypocalcemia.^23^ The fact that that autotransplantation predicted late hypocalcemia may also reflect that this variable represents a more challenging resection and increased likelihood of vascular disruption to other parathyroid glands. Furthermore, subtotal parathyroidectomy was associated with higher rates of early hypocalcemia compared with single gland excision, which may represent the time it takes for the remaining partial gland to compensate for total parathyroid function in the immediate postoperative period. We did not see differences for late hypocalcemia, reflecting restoration of parathyroid function in the remnant. Early postoperative PTH levels (within 7 days) were not significantly associated with early or late hypocalcemia in our models. Based on these results, the laboratory value alone does not appear to be a strong predictor for hypocalcemia and should be used with caution in these populations.

Postoperatively, neither amount nor type of calcium supplementation was associated with lower rates of hypocalcemia. Interestingly, while calcium citrate is known to have superior bioavailability in RYGB,^20^ only 25% of patients with RYGB anatomy were prescribed calcium citrate postoperatively compared to 68% prescribed calcium carbonate. Given that this analysis was based on the encounter for the cervical procedure, prescribing patterns likely reflect the endocrine surgeon practice rather than bariatric surgery. This is an area in which increased awareness could impact endocrine surgery prescribing patterns to increase the proportion of patients receiving calcium citrate. However, even when prescribed postoperatively, calcium citrate was not associated with a lower risk of hypocalcemia, indicating that this alone may not be a curative factor. The amount of elemental calcium prescribed was in fact *positively* correlated with early hypocalcemia in the multivariable model, suggesting that patients experiencing hypocalcemia were prescribed increasing quantities of calcium without resolution. This reinforces the significance of our findings on preoperative calcium and potential for optimization.

Our analysis of secondary outcomes revealed that 5.4% of patients required readmission due to hypocalcemia, similar to rates of hypocalcemia requiring intervention in the literature.^10^ While GJ anatomy was associated with prolonged LOS and readmission overall, most were not readmitted for hypocalcemia, which may reflect underlying comorbidities in this population. These findings may inform decision making regarding the hospital setting in which to operate on these higher risk patients, impacting expected healthcare utilization. Fortunately, overall, most cases of hypocalcemia in this study could be managed outpatient. These findings have implications for the importance of patient counseling and outpatient surveillance.

### Limitations

Our study is limited by the retrospective design, which is dependent on chart review and data available in the electronic medical record. Retrospective studies cannot assume causality, and we are limited to reporting associations. A strength of this study is the multi-institutional design that enabled us to include a larger and more diverse patient population. Every effort was made to gather complete patient information, and we were cautious when comparing laboratory values across institutions to evaluate them in the context of the defined laboratory thresholds. In addition, given the high incidence of the primary outcomes, the odds ratios predicated in univariate and multivariable models will likely overpredict the relative risk among variables of interest. Therefore, we are measured when interpreting the magnitude of the OR, but the directionality and significance of these findings contribute to our understanding of the population of patients with prior GJ undergoing cervical procedures. When interpreting results related to medications prescribed, we are also limited by patient compliance in both taking medications and reporting medication lists, which may impact our interpretation of use of calcium and antacids. Future studies could include more preoperative diagnostic data including thyroid comorbidities, such as Graves’ disease.

## Conclusions

Patients with histories of nonbariatric GJ or bariatric RYGB experience high rates of early and late hypocalcemia following parathyroidectomy and total thyroidectomy. The highest risk of hypocalcemia occurred among those with a history of GJ or RYGB undergoing total thyroidectomy. A history of nonbariatric GJ was also associated with longer LOS and 30-day readmission. These findings demonstrate that a history of any form of duodenal bypass may independently put patients at higher risk of postoperative hypocalcemia following cervical procedures. Surgeons should be aware of this patient population with implications for preoperative counseling, postoperative surveillance, and calcium supplementation. Notably, traditional management with calcium citrate and higher doses of elemental calcium postoperatively were not protective against hypocalcemia, highlighting an area for needed research to help mitigate hypocalcemia in this at-risk population. As preoperative calcium levels were protective against postoperative hypocalcemia, preoperative optimization with calcium supplementation is one potential mitigative strategy to further study in this population.
